# Editorial: Cell death pathways in bronchopulmonary dysplasia: mechanisms and therapeutic targets

**DOI:** 10.3389/fphys.2026.1955403

**Published:** 2026-10-13

**Authors:** Siyi Shi, Yequan Xu, Renqiang Yu, Stefano Nobile, Shanyu Jiang

**Affiliations:** 1 Affiliated Women’s Hospital of Jiangnan University, Wuxi Maternity and Child Health Care Hospital, Wuxi, China; 2 Department of Public Health and Preventive Medicine, Wuxi School of Medicine, Jiangnan University, Wuxi, China; 3 Department of Mother, Child and Public Health, Fondazione Fondazione Policlinico Universitario A. Gemelli IRCCS, Rome, Italy

**Keywords:** bronchopulmonary dysplasia, cellular senescence, mechanism-based therapy, mitochondrial dysfunction, programmed cell death

## 1 Introduction

Bronchopulmonary dysplasia (BPD) begins when a lung that should still be building alveoli and its microvascular network must adapt prematurely to an extrauterine environment. Hyperoxia, mechanical ventilation, and infection or inflammation can each add injury, but the central problem is the loss of coordinated development. This helps explain why BPD is associated not only with simplified alveoli, but also with persistent pulmonary vascular and respiratory consequences (Thebaud et al., 2019). Apoptosis is one of the earliest and most extensively characterized forms of programmed cell death implicated in BPD pathogenesis (De Paepe et al., 2005). In contrast, pyroptosis and ferroptosis have emerged more recently as potential contributors, with current evidence derived predominantly from experimental models and limited translational or human studies (Xu et al., 2023; Ruan et al., 2025). The key question is therefore not simply which insult occurs first, but how repeated stress changes the capacity of immature lung cells to survive, repair, and continue developing.

The articles in this Research Topic can be read as successive parts of that question. They move from cellular stress and cell fate, to ways of detecting these processes in infants and locating them within the lung, and finally to the conditions required before a mechanistic observation can become a treatment. This framework deliberately distinguishes established pathological features from emerging observations and from findings limited to cell or animal models. Together, the contributions outline a developmental model of BPD rather than a collection of independent pathways.

## 2 Mechanistic insights

As research into Bronchopulmonary dysplasia (BPD) has advanced, the understanding of its pathogenesis has evolved beyond single-factor explanations. Oxidative stress, immune and inflammatory dysregulation, impaired alveolar and pulmonary vascular development, and multiple forms of programmed cell death are now recognized as interconnected pathological processes that shape the BPD phenotype (Thebaud et al., 2019; Deng et al., 2023). However, despite growing recognition of these pathological processes, a unifying framework explaining how they converge to drive BPD progression has remained elusive. Meanwhile, mitochondrial dysfunction is increasingly recognized as a common feature accompanying these pathological processes (Ratner et al., 2009). Recent studies have underscored the integrative role of mitochondrial dysfunction in linking oxidative stress, inflammatory responses, cell death, and impaired lung development (Zhang et al.). This does not establish mitochondrial dysfunction as a universal upstream cause across human BPD phenotypes. It does, however, provide a useful organizing hypothesis: when mitochondrial reserve is inadequate, the developing lung may be less able to contain stress within a reparative response. Defining whether this process is causal, in which cells it occurs, and when it becomes actionable remains a core Research Topic.

Cell fate is one consequence of this loss of reserve. A recent review describes potential crosstalk among apoptosis, necroptosis, pyroptosis, and ferroptosis through shared molecular regulators (Jiang et al.). The available literature suggests that they may overlap, compete, or emerge at different points in injury, but direct evidence for specific switching events and cross-cellular communication networks in BPD remains limited (Huang et al., 2025; Jiang et al.). Among these cell-fate responses, the emergence of lytic forms of cell death may reflect impaired apoptotic resolution under converging metabolic and clearance constraints (Marega and Chao). This interpretation is plausible but remains a hypothesis that requires cell-specific and time-resolved testing in BPD-relevant models.

Beyond the mechanistic view centered on mitochondrial dysfunction and cell death, a recent study implicates a novel Noggin–BMP4–p53/p21 signaling axis in hyperoxia-induced endothelial senescence. In one study of hyperoxia-exposed human pulmonary microvascular endothelial cells and neonatal rats, increased Noggin expression, reduced BMP4 expression, and activation of p53/p21 were associated with a senescent phenotype; Noggin silencing attenuated these changes *in vitro*. These findings implicate the Noggin–BMP4–p53/p21 axis in hyperoxia-associated cellular senescence (Zhang et al.). However, whether senescence precedes or predisposes cells to other forms of cell death, and whether this pathway causally contributes to BPD, remain to be established.

Seen together, these mechanistic contributions shift attention from damage to developmental trajectory. Oxidative stress may reduce cellular resilience; while altered mitochondrial homeostasis may influence whether epithelial, endothelial, immune, and stromal cells adapt, arrest, or die. The eventual phenotype is impaired alveolar-vascular development. The strength of this framework lies in the questions it creates: which cells are affected first, which responses are reversible, and which molecular changes identify infants at risk of BPD before structural arrest of lung development is established.

## 3 Methodological innovation

Bridging mechanistic insights and clinical observations is another major focus. A retrospective cohort study found that lower levels of total triiodothyronine, total thyroxine, free triiodothyronine, and free thyroxine at 2 and 4 weeks after birth were associated with more severe BPD in extremely and very preterm infants. These findings suggest that serial thyroid hormone measurements may warrant further evaluation as candidate markers of BPD severity, although their predictive value requires validation in larger prospective cohorts (Ma et al.). In another clinical study, a retrospective cohort study in children with Mycoplasma pneumoniae pneumonia identified age, C-reactive protein, white blood cell count, season, and IgM and IgA levels as independent predictors of pleural effusion (Liu and Cai). Although not directly related to BPD, this study illustrates a broader clinical approach within the Research Topic: combining routinely available clinical and laboratory variables to identify children at increased risk of respiratory complications.

However, advancing mechanistic studies and validating multiple biomarkers will continue to depend on access to high-quality specimens that reflect the local pulmonary environment. Though tracheal aspirates are considered ideal specimens for investigating local pulmonary pathology, their collection is restricted to intubated infants, limiting patient eligibility and the availability of appropriate controls (Kimble et al., 2022). A small exploratory study used high-resolution proteomics to compare paired nasopharyngeal and tracheal aspirates from five preterm infants. The substantial overlap in their proteomic profiles suggests that nasopharyngeal aspirates may provide a less invasive source of respiratory biomarker information, although their equivalence to tracheal aspirates and their utility for BPD biomarker discovery require validation in larger cohorts (Garrido-Munoz et al.).

The establishment of clinical predictive indicators provides a foundation for the early identification of infants with BPD. Nevertheless, pulmonary pathological alterations may vary across different regions of the lung, and conventional analyses do not always preserve this spatial context. To address this limitation, an integrated imaging workflow combining micro-CT-based three-dimensional reconstruction with high-resolution light microscopy and immunofluorescence has been extended to incorporate cell-specific molecular information. Using prosurfactant protein C (pro-SP-C) as a cellular marker, the workflow demonstrated the feasibility of localizing cell-specific information within the three-dimensional branching architecture of the lung (Labode and Mühlfeld). This approach provides a methodological basis for future investigations into the regional distribution of pathological alterations in BPD and other pulmonary diseases.

## 4 Therapeutic exploration

A developmental map should guide therapeutic testing. It suggests three linked aims: reduce the initial cellular stress, preserve reversible cell states, and support recovery once structural injury has occurred. Therapeutic strategies directed at these aims remain largely preclinical and should not be interpreted as substitutes for established supportive and preventive care. Translation will require evidence of target engagement, developmental safety, appropriate dose and timing, and meaningful clinical benefit.

Interventions directed at mitochondrial homeostasis, antioxidant defense, or excessive mitochondrial fission may therefore represent potential strategies for limiting early cellular injury. Molecular hydrogen illustrates both the opportunity and the limitations of this approach. In hyperoxia-exposed neonatal rats and RLE-6TN cells, hydrogen was associated with improved lung morphology, less apoptosis and endoplasmic-reticulum stress, and altered AHR-CPEB4 signaling (Liang et al.). These findings warrant replication and mechanistic refinement, including dose and delivery studies; they currently do not support clinical adoption in preterm infants.

When cells enter inflammatory cell-death programs or a senescent state, therapeutic intervention may become more complex. RIPK1- and NLRP3-directed approaches, as well as modulation of the Noggin–BMP4–p53/p21 axis, have been proposed or explored in preclinical settings as candidate therapeutic targets for BPD (Jiang et al.; Zhang et al.). However, their therapeutic value in BPD remains unknown. Any intervention will need to account for developmental timing, cell specificity, pathway compensation, and the possibility that suppressing a stress response may also interfere with normal lung maturation.

When BPD progresses to the stage of structural injury, regenerative strategies may offer the potential to support alveolar and pulmonary vascular repair. MSCs and MSC-derived extracellular vesicles, including exosomes, have improved selected features of experimental BPD models, and proposed mechanisms include paracrine effects and transfer of mitochondrial components (Jiang et al.). A phase I dose-escalation study of selected umbilical-cord-blood-derived MSCs in nine preterm infants supported feasibility and short-term safety, whereas a subsequent randomized phase II trial did not significantly reduce the primary composite outcome of death or moderate-to-severe BPD (Chang et al., 2014; Ahn et al., 2021). MSC-derived extracellular vesicles and exosomes remain predominantly preclinical approaches. Further multicenter studies are therefore required to define efficacy, developmental safety, dose, route, timing, and appropriate product characteristics before these strategies can be considered for routine BPD care.

The translational challenge is thus not a shortage of targets. It is the need to match each target to a defined stage of disease and a measurable biological state. For many of the emerging mechanisms and therapeutic strategies discussed here, the evidence base remains dominated by cultured cells, animal models, small retrospective cohorts, and exploratory studies. Prospective multicenter cohorts can determine which systemic and airway signals consistently precede structural injury and are associated with subsequent disease progression. Reproducible, cell-specific models can then test whether the mechanisms associated with these signals causally influence developmental outcomes. Clinical trials can ultimately assess whether mechanism-guided interventions improve meaningful outcomes without disrupting normal lung development.

This Research Topic frames BPD as a disorder of developmental resilience. Its mechanistic, methodological, and therapeutic contributions can be integrated into a proposed developmental framework in which cellular stress reduces reserve, loss of reserve influences cell fate, and altered cell fate disrupts alveolar and pulmonary vascular development. The translational task is to determine which steps in this proposed pathway are biologically valid, clinically measurable, and safe to target in the developing lung.
